# Involvement of both Cervical Lymph Nodes and Retropharyngeal Lymph Nodes has prognostic value for N1 patients with Nasopharyngeal Carcinoma

**DOI:** 10.1186/1748-717X-9-7

**Published:** 2014-01-06

**Authors:** Qi Shi, Chunying Shen, Lin Kong, Xiaoshen Wang, Jianhui Ding, Yunsheng Gao, Tingting Xu, Chaosu Hu

**Affiliations:** 1 Department of Radiation Oncology, Cancer Hospital of Fudan University, 270 Dong An Road, Shanghai, China; 2 Department Diagnostic Radiology, Cancer Hospital of Fudan University, Shanghai, China

**Keywords:** Nasopharyngeal carcinoma, Lymph nodes, Prognosis

## Abstract

**Background:**

The N1 definition of 2010 UICC/AJCC staging system for nasopharyngeal carcinoma (NPC) covers quite a large range of nodal pattern. The objective of this research is to investigate prognostic value of lymph nodes related factors including involvement of both cervical lymph nodes (CLNs) and retropharyngeal lymph nodes (RLNs) or not, size and number of cervical lymph nodes (CLNs) in N1 patients with NPC.

**Methods:**

142 newly diagnosed non-metastatic N1 patients with NPC, staged according to the 2010 AJCC staging system for NPC were retrospectively enrolled. All patients had undergone contrast-enhanced magnetic resonance imaging (MRI), and received radiotherapy, with or without chemotherapy as their primary treatment.

**Results:**

The median follow-up was 48 months. The 5-year local recurrence-free survival (LFS), nodal recurrence-free survival (NFS), local-regional recurrence-free survival (LRFS), distant metastasis-free survival (DMFS), progression-free survival (PFS), and overall survival (OS) of the whole group were 82.3%, 83.0%, 81.0%, 82.1%, 75.3% and 89.8%, respectively. In univariate analysis, patients with both CLNs and RLNs involvement showed a significant lower DMFS, PFS and LRFS than the rest patients (p = 0.004 p = 0.003 and p = 0.034, respectively). Neither size nor number of CLNs affected the survival. In multivariate analysis, involvement of both CLNs and RLNs was an independent prognostic factor for DMFS and PFS (p = 0.019, p = 0.019), but there was no enough evidence confirming its prognostic value for LRFS (p = 0.051).

**Conclusions:**

For N1 patients with NPC, involvement of both RLNs and CLNs may be a potentially prognostic factor for distant metastasis and disease progression. The N stage for N1 patients with involvement of both cervical lymph nodes and retropharyngeal lymph nodes might need to be deliberated.

## Background

Nasopharyngeal carcinoma (NPC) is one of the most endemic head and neck cancer in Southeast Asia and East Asia. The incidence of NPC in Southern China is approximately 30 to 80 per 100,000 people per year [1]. Radiotherapy is the common recognized treatment modality for nasopharyngeal carcinoma. The use of IMRT and the combination with chemotherapy have improved the locoregional control of NPC, while distant metastasis and recurrence are the main failures after treatment [2,3].

Tumor-nodal-metastasis (TNM) system for nasopharyngeal carcinoma (NPC) is helpful in predicting prognosis, facilitating treatment planning, and exchanging information between different centers. Generally speaking, the T-classification can predict local control, while the N-classification can predict neck and distant control. Union for International Cancer Control/American Joint Committee on Cancer (UICC/AJCC) TNM staging system for NPC is the most common recognized staging system. The most recently published seventh edition of the UICC/AJCC staging system have recommended several changes in the definition of T-classification and N-classification to the sixth edition, and one of the important improvement was upgrading patients with involvement of retropharyngeal lymph nodes (RLNs) only and without cervical lymph node (CLN) metastasis to N1 [4,5].

According to the 7^th^ edition UICC/AJCC staging system, N1 classification for NPC is defined as following: unilateral lymph nodes, 6 cm or less, above the supraclavicular fossa, and/or retropharyngeal lymph nodes, 6 cm or less (unilateral or bilateral) [5]. Thus, the N1 definition of UICC/AJCC staging system covers quite a large range of nodal patterns, which may includes patients with or without retropharyngeal lymph nodes, patients with or without cervical lymph nodes, patients with cervical lymph nodes of different sizes, and patients with single or multiple cervical lymph nodes. It is reasonable to hypothesize that N1 patients with different nodal characteristics may have different prognosis.

Few studies had ever focused on the possibly different outcomes in NPC patients with N1 disease. This study was conducted to find out whether long-term prognosis differs among N1 patients with different nodal characteristics, in order to suggest whether detailed classification or improvement is needed in the definition of N1 in 7^th^ edition UICC/AJCC staging system for NPC, and to more effectively guide future treatment.

## Methods

### Patients

From November 2007 through November 2009, 180 consecutive patients were enrolled in this study and were retrospectively reviewed. The inclusion criteria were as following: (1) biopsy-proven nasopharyngeal carcinoma; (2) no previous treatment including chemotherapy, radiotherapy, surgery, etc.; (3) with pretreatment magnetic resonance imaging (MRI) of the nasopharynx and neck; (4) N1 classification according to the definition of the 7^th^ edition UICC/AJCC staging system for NPC, which dominantly depends on MRI; (5) from 18 to 70 years old; (6) receiving radiotherapy in our institute. The exclusion criteria were as follows: (1) with distant metastasis proven by clinical or radiologic evidence; (2) previously diagnosed as other head and neck cancers and had received surgery, radiotherapy, or chemotherapy before.

This study was approved by the Research Ethics Committee of Fudan University Shanghai Cancer Hospital and was performed in accordance with the ethical standards laid down in the 1964 Declaration of Helsinki and all subsequent revisions.

### Pretreatment evaluation

Pretreatment evaluation were composed of complete medical history, physical examination, indirect or fiberoptic endoscopic examination of nasopharynx, biopsy of the neoplasm in nasopharynx, MRI scans of nasopharynx and neck, chest CT or X-ray, abdominal sonography, and bone scans for T3-T4 and symptomatic patients.

All patients were staged according to the 7^th^ edition of AJCC staging system [5]. Cervical lymph nodes were defined as metastatic if the shortest axial diameter of jugulodigastric lymph node was ≥11 mm, or the shortest axial diameter of other lymph nodes was ≥10 mm, or there was a group of three or more lymph nodes in critical size [6,7]. The lateral RLNs were considered metastatic with their shortest diameter ≥5 mm. Any visible node in the median retropharyngeal group was considered malignant [8,9]. Moreover, the presence of extracapsular spread and central necrosis were also signs of metastasis [6]– [9].

### Radiotherapy

#### Intensity-Modulated Radiation Therapy (IMRT)

Patients were immobilized in the supine position with a thermoplastic mask. CT simulation was performed after immobilization, obtaining 3-mm slices from the anterior clinoid process to the hyoid bone, and 5-mm slices from the hyoid bone to 2 cm below the sternoclavicular joint. The gross tumor volume (GTV) included primary tumor and enlarged RLNs. The clinical target volume (CTV) included bilateral coverage of levels II, III, VA and RLNs. The CTV should also cover the entire nasopharynx, parapharyngeal space, clivus, base of skull, pterygoid fossa, posterior half of ethmoidal sinus, inferior sphenoid sinus, and posterior third of nasal cavity and maxillary sinuses. A margin of 3–5 mm around GTV and CTV should be added to account for the patient motion and set-up error. The total dose to primary tumor was 66 Gy in 30 fractions for T1 or T2 disease, 70.4 Gy in 32 fractions for T3 or T4 lesion. A total dose of 60 Gy was delivered to the CTV in 30–32 fractions.

#### Conventional two-dimensional radiotherapy (2D-RT)

For patients treated with 2D-RT, immobilization was the same as that used for IMRT. Two lateral opposed fields were used to irradiate the nasopharynx and upper neck, followed by the shrinking-field technique to limit dose to spinal cord. A Boost of 4–6 Gy was routinely delivered to the skull base in patients with involvement of skull base or intracranial extension. An anterior field was used to treat the neck. In patients with no cervical lymph node metastasis, only the upper neck was treated, including levels II, III and VA. In others the whole neck received radiation, including levels II, III, IV, V, according to the EORTC and RTOG consensus guideline. The accumulated dose to the primary tumor was 70–76 Gy with 2 Gy per fraction. The dose to uninvolved neck regions ranged from 50–62 Gy with 1.8 to 2 Gy per fraction.

### Boost

Residual disease diagnosed by clinical examination or MRI was boosted. Superficial lesion was treated by brachytherapy, with a dose of 6–16 Gy in one or two weekly fractions. More advanced disease was treated by small field external-beam radiotherapy, including IMRT, 3D-RT, 2D-RT and stereotactic radio-surgery. The boost dose was 6–8 Gy.

### Chemotherapy

There were several regimens of neoadjuvant chemotherapy and adjuvant chemotherapy in our institution, including PF, TPF and GP. The TPF protocol consisted of docetaxel 75 mg/m^2^ IV on day 1, cisplatin75 mg/m^2^ IV on day 1, and 5-fu 500 mg/m^2^ d continuously IV on day1–5. The PF protocol consisted of cisplatin 75 mg/m^2^ IV on day 1 and 5-fu 500 mg/m^2^ d continuously IV on day 1–5. The GP regimen included cisplatin 75 mg/m^2^d IV on day 1 and gemcitabine 1000 mg/m^2^ IV on day 1, 8. Regimens were repeated every 3 weeks for 2 cycles as neoadjuvant chemotherapy. This was followed by cisplatin 40 mg/m^2^ IV weekly during radiation. For patients who received adjuvant chemotherapy, regimens were repeated every 3–4 week for 3 cycles.

### Follow-up

The follow-up period ranged from the first day of the date of initiation of treatment until death or the last visit. The median follow-up period was 48 months (range, 2 – 66 months).

Patients were followed every 3 months in the first to second year, then every 6 months in the third to fifth year and once a year thereafter. In each visit, medical history, physical examination and nasopharyngoscopy were performed. Nasopharyngeal MRI was performed 3 months and 1 year after completion of radiotherapy, and every 6 months in the second to fifth year, and then yearly thereafter. The following tests were done at least every year: chest CT or X-ray, abdominal sonography, and bone scan when clinically indicated. Late toxicities were evaluated according to the toxicity criteria of the RTOG [10].

### Statistical analysis

All analyses were performed using the SPSS software, version 19.0. The actuarial rates were estimated by the Kaplan–Meier method, and the survival curves were compared with the log-rank test [11]. The following endpoints were assessed: overall survival (OS), local recurrence-free survival (LFS), nodal recurrence-free survival (NFS), local-regional recurrence-free survival (LRFS), distant metastasis-free survival (DMFS), and progression-free survival (PFS). All the endpoints were defined as the interval from the date of initiation of treatment to the date of the failure or death, or last follow-up. Multivariate analyses with the Cox proportional hazards model [12] were used to test for independent significance by backward elimination of insignificant explanatory variables of the different parameters.

## Results and discussion

### Patients and treatments

Between 2007 and 2009, a total of 180 NPC patients with N1 disease were enrolled in this study. Among them, 1 patient failed to complete radiotherapy due to Grade 3 mucositis and poor general condition. The pretreatment nasopharyngeal MR images of 37 patients were not available because of image-preserving reason. Thus, there were 142 assessable patients. Clinical characteristics were listed in Table 1. The median age was 50 years old (range: 23–70 years). Of patients with stage III/IV disease, 98.2% (56/57) received chemotherapy. Of patients with stage II disease, 82.35% (70/85) received chemotherapy. 35 patients were treated by 2D-RT, and the rest 107 patients were treated by IMRT. 85 patients belonged to T1-T2 stage, and 57 belonged to T3-T4 stage.

**Table 1:** Patient characteristics

		**Number**	**Percentile (%)**
Sex	Male	106	74.6
	Female	36	25.4
Age	Median	50	
	Range	23-70	
Chemotherapy	Yes	126	88.7
	No	16	11.3
Radiotherapy	2D-RT	35	24.6
	3D-CRT/IMRT	107	75.4
T Stage	T1-2	85	59.9
	T3-4	57	40.1
RLN	RLN+	109	76.8
	RLN-	33	23.2
CLN	CLN+	110	77.5
	CLN-	32	22.5

RLN indicates retropharyngeal lymph node.

CLN indicates cervical lymph node.

### Survival and patterns of failure

With a median follow-up of 48 months (range, 2 – 66 months), a total of 9 local recurrences, 8 nodal recurrences, 15 distant metastasis, and 12 death were observed. The main failure was distant metastasis. Among the 15 patients developing distant metastasis, 14 had both cervical lymph nodes and retropharyngeal lymph nodes involvement, and the rest one had only retropharyngeal lymph node metastasis. The patterns of failure were listed in Table 2, and the common sites of distant failure were liver, lung and bone. The 5-year local recurrence-free survival (LFS), nodal recurrence-free survival (NFS), local-regional recurrence-free survival (LRFS), distant metastasis-free survival (DMFS), progression-free survival (PFS), and overall survival (OS) of the whole group were 82.3%, 83.0%, 81.0%, 82.1%, 75.3% and 89.8%, respectively.

**Table 2:** Patterns of failure

**Failure**	**Frequency**	**%**
Local recurrence	9	6.3
Nodal recurrence	8	5.6
Local & nodal recurrence	5	3.5
Distant metastasis	15	10.6
Local recurrence & distant metastasis	1	0.7
Local & nodal recurrence & distant metastasis	1	0.7
Death	12	8.5

### Patterns of lymph nodes involvement

ALL of the 142 NPC patients were assured to be classified into N1 category according to the 7^th^ edition UICC/AJCC staging system. The patterns of metastatic lymph nodes involvement were presented in Table 3. Among the population, 110 patients had metastatic cervical lymph nodes (CLNs), and the rest 32 patients had no cervical lymph nodes but with unilateral or bilateral retropharyngeal lymph nodes (RLNs). 77 patients had both RLNs and CLNs involvement, and the 33 patients had CLNs only. The longest diameters of CLNs in 65 patients were less than 3 centimeters. 61 patients had more than 1 CLNs, and the rest 49 patients had only single CLN. Only 6 patients had CLNs in level IV, while the CLNs of other patients were all in level Ib, II, and III. Among the 109 patients had metastatic RLNs, 80 were with unilateral RLN metastasis, and 29 were with bilateral RLN metastasis.

**Table 3:** Patterns of lymph nodes involvement

	**Number**
CLN-/RLN+	32
CLN+/RLN-	33
CLN + & RLN+	77
Longest diameter of CLN ≤ 3 cm	65
3 cm < Longest diameter of CLN ≤ 6 cm	45
Single CLN metastasis	49
Multiple CLNs metastases	61
CLN in lower neck	6
Unilateral RLNs metastasis	80
Bilateral RLNs metastasis	29

RLN indicates retropharyngeal lymph node.

CLN indicates cervical lymph node.

### Univariate and multivariate analyses

The Kaplan-Meier method was used to estimate LFS, NFS, LRFS, DMFS, PFS, and OS for different nodal status, including patients with both CLNs and RLNs versus the rest, longest diameter of CLN 3-6 cm versus ≤ 3 cm, and single CLN versus multiple CLN.

**Figure 1:**
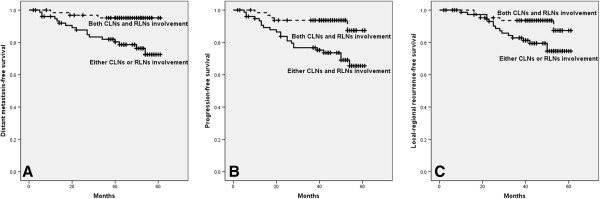
**DMFS, PFS and LRFS of patients with different nodal characteristics. (A)** Patients with both CLNs and RLNs showed lower DMFS than those with either CLNs or RLNs ( p=0.004). **(B)** Patients with both CLNs and RLNs showed lower PFS than those with either CLNs or RLNs (p=0.003). **(C)** Patients with both CLNs and RLNs showed lower LRFS than those with either CLNs or RLNs ( p=0.034).

Among the 142 patients, 32 patients with only RLNs (no CLN) and 33 patients with only CLNs (no RLN) showed similar prognosis in LFS, NFS, LRFS, DMFS, PFS and OS (96.4% vs 75.5%, p = 0.181; 96.4% vs 75.8%, p = 0.166; 96.4% vs 75.8%, p = 0.166; 96.7% vs 93.8%, p = 0.636; 96.7% vs 75.8%, p = 0.177; 96.4% vs 93.8%, p = 0.630). However, those with both CLNs and RLNs involvement showed a significant lower DMFS, PFS and LRFS than those with only RLNs and with only CLNs (Figure 1). No significant difference was observed in LFS, NFS, and OS between the two groups (Table 4). As T stage and concurrent chemotherapy may affect patients’ survival, independent-samples t-test was conducted to make sure that the distribution of T stage and that of patients received concurrent chemotherapy were well balanced between the two groups (p = 0.727, p = 0.117).

**Table 4:** Univariate analysis of lymph nodes related variables and other clinical factors for 5-year survival

**Variable**	**Patient no.**	**LFS (%)**	**p**	**NFS (%)**	**p**	**LRFS (%)**	**p**	**DMFS (%)**	**p**	**PFS (%)**	**p**	**OS (%)**	**p**
Both CLNs and RLNs													
Yes	77	77.2	0.068	78.4	0.112	74.6	**0.034**	72.5	**0.004**	65.5	**0.003**	85.3	0.119
No	65	87.1		87.3		87.3		95.2		87.4		95.1	
Size of CLNs													
CLN ≤ 3 cm	66	81.0	0.988	83.5	0.940	80.5	0.966	79.8	0.426	74.0	0.448	88.8	0.853
CLN 3-6 cm	44	74.1		74.0		72.2		76.0		64.7		87.2	
Number of CLNs													
Single CLN	62	73.9	0.328	74.1	0.174	70.7	0.154	71.5	0.240	60.3	0.132	85.6	0.562
Multiple CLNs	48	84.8		86.4		84.8		86.7		81.2		90.5	
Age													
<50	74	83.8	0.858	84.8	0.683	82.4	0.881	81.3	0.649	77.4	0.802	89.9	0.542
>50	68	81.0		81.3		79.8		83.0		74.0		88.9	
Sex													
Male	106	79.5	0.215	80.6	0.215	78.9	0.355	77.0	0.066	70.6	0.114	86.9	0.123
Female	36	89.3		89.0		86.3		94.3		86.3		97.1	
Radiotherapy													
2D-RT	35	77.5	0.263	80.5	0.526	75.2	0.207	66.7	**0.009**	62.4	**0.027**	83.0	0.160
IMRT	107	83.2		83.0		82.1		89.5		80.5		92.7	
Chemotherapy													
Yes	16	92.3	0.379	84.6	0.995	84.6	0.815	85.7	0.937	77.9	0.950	92.3	0.765
No	126	80.7		82.4		80.2		81.5		74.4		89.4	
Pathology													
WHO II	37	76.2	0.238	75.1	0.167	73.0	0.171	79.3	0.347	68.7	0.183	88.1	0.473
WHO III	105	84.3		85.5		83.5		82.6		76.9		90.4	
T Stage													
T1-T2	85	89.6	**0.032**	88.5	0.165	87.3	0.097	87.8	**0.005**	81.6	**0.012**	94.7	**0.048**
T3-T4	57	72.5		75.7		72.6		72.5		65.4		82.4	

Bold numbers, indicated there was statistically significant difference (p value < 0.05).

RLN indicates retropharyngeal lymph node.

CLN indicates cervical lymph node.

2D-RT indicates Conventional two-dimensional radiotherapy and IMRT indicates Intensity-Modulated Radiation Therapy.

LFS, NFS, LRFS, DMFS, PFS and OS indicate for local recurrence-free survival, nodal recurrence-free survival, local-regional recurrence-free survival, distant metastasis-free survival, progression-free survival and overall survival.

We also performed a subgroup analysis by T stage. In patients with T3-T4 disease, the significant difference in DMFS and PFS (90.9% vs 59.5%, p = 0.021; 79.5% vs 54.7%, p = 0.025) between the two groups still existed and a relatively lower LRFS (79.5% vs 65.9%) was observed in patients with both metastatic CLNs and RLNs in spite of no statistical difference. In those with T1-T2 disease, we could also see an increasing rate of DMFS, PFS and LRFS in patients with either CLNs or RLNs involvement over those with both CLNs and RLNs (97.6% vs 82.1%; 95.1% vs 73.6%; 94.9% vs 80.9%).

Neither the size nor number of CLNs was the factor affecting the survival of patients. No significant difference were found in LFS, NFS, LRFS, DMFS, PFS and OS in patients with CLN ≤ 3 cm vs those with CLN > 3 cm, and in patients with single CLN vs those with multiple CLN (Table 4). After stratified by T classification, size and number of CLNs still did not associate with the above endpoints.

In multivariate analysis, involvement of both CLNs and RLNs or not, T stage, technique of radiotherapy and sex, which were proved by univariate analysis to have the potential affecting survival rate were included in the Cox proportional hazard model. The results showed that involvement of both CLNs and RLNs was an independent prognostic factor for DMFS and PFS (p = 0.019, p = 0.019), but there was no enough evidence confirming its prognostic value for LRFS (p = 0.051) (Table 5).

**Table 5:** Summary of multivariate analysis of factors in patients with N1 disease

**Endpoint**	**Factor**	**B**	**P value**	**Exp (B)**	**95% CI for Exp (B)**
DMFS	Both CLNs and RLNs involvement	-1.498	**0.019**	0.224	0.064-0.783
	T stage	-1.374	**0.005**	0.253	0.096-0.666
	Technique of radiotherapy	0.978	**0.035**	2.660	1.072-6.603
	Sex	1.310	0.081	3.705	0.853-16.093
PFS	Both CLNs and RLNs involvement	-1.179	**0.019**	0.307	0.115-0.825
	T stage	-1.046	**0.010**	0.351	0.159-0.776
	Technique of radiotherapy	0.773	0.054	2.167	0.988-4.751
LRFS	Both CLNs and RLNs involvement	-1.002	0.051	0.367	0.134-1.003
	T stage	-0.671	0.129	0.511	0.215-1.215

Bold numbers, indicated there was statistically significant difference (p value < 0.05).

RLN indicates retropharyngeal lymph node.

CLN indicates cervical lymph node.

LRFS, DMFS, PFS indicate for local-regional recurrence-free survival, distant metastasis-free survival and progression-free survival.

## Discussion

Our study is the first research focusing on the outcome and prognosis of NPC patients with N1 disease according to the 7^th^ edition UICC/AJCC staging system. And the stages of patients in our study were all based on magnetic resonance imaging, which is the commonly recognized best modality for staging locoregional NPC [13]– [15] and allows a more accurate assessment of the extent of primary tumor and lymph node status than computerized tomography (CT) because of its multiplanar capacity and improved tissue contrast [14].

The 5-year LFS, NFS, LRFS, DMFS, PFS, and OS were 82.3%, 83.0%, 81.0%, 82.1%, 75.3% and 89.8%, respectively. The results of our study are comparable to other series [16]– [18]. As 24.6% (35/142) of our patients enrolled in were treated by 2D-RT, the 5-year LFS, DMFS, PFS are slightly lower than those of Sun et al. treated with 3D-CRT and IMRT (90%, 86% and 77%, respectively) [16]. The overall survival of 89.8% is remarkably better than 64.4–67.9% of He et al. [17] and Gao et al. [18] treated with 2D technique.

In our study, no significant difference was observed in all survival related endpoints between patients with CLN > 3 cm and those with CLN ≤ 3 cm, and between patients with single CLN and multiple CLN, indicating that size and number of metastatic CLNs might not be the valuable factors which affect N1 patients’ survival.

Quite a large amount of studies have compared different endpoints between N0 patients (6^th^ edition) with RLNs and N0 patients (6^th^ edition) without RLN, and between N0 patients (6^th^ edition) with RLNs and N1 patients (6^th^ edition). The results reported by these studies were similar: in N0 (6^th^ edition) patients, involvement of RLNs was an independent predictor for DMFS [19]– [21]. Ma’s study showed that RLNs metastases also predict for OS and LRFS [19]. And all these studies found no significant difference in hazard ratio for distant metastasis and death between patients with N0 disease with RLNs involvement and patients with N1 disease (6^th^ edition) [19]– [21]. Nevertheless, few studies has focused on the probably prognostic value of metastatic lymph nodes pattern in N1 patients. In our study, of the 15 patients developing distant metastasis, 14 patients were with both CLNs and RLNs involvement. And among the 142 patients, those with either RLNs or CLNs involvement showed a significantly better DMFS, PFS and LRFS than those with both CLNs and RLNs patients. Multivariate analysis confirmed that those with both CLNs and RLNs metastases had a significantly higher risk for distant metastasis and disease progression. This result should be considered reasonable, with historical data nearly all concluding retropharyngeal lymph node metastasis as a prognostic factor for DMFS in N0 patients (6^th^ edition).

Anatomically, RLNs are located within the retropharyngeal space. The pharyngeal constrictor muscles bound this space anteriorly, the pre-vertebral fascia posteriorly, the carotid sheath laterally, the skull base superiorly and inferiorly the space continues to the level of vertebra C3 at its caudal extent [22]. The involvement of RLNs in nasopharyngeal carcinoma is frequent, with a high incidence of RLNs metastases ranges from 63.4%-94% [8,20,23,24] assessed by MRI. Actually, several clinical studies showed that RLNs represent first-echelon lymph nodes typically involved in NPC [8,24,25].

The publishing of 2010 AJCC/UICC staging system (7^th^ edition) have upgraded NPC patients with RLNs metastases with no other CLN to N1, and before that, the staging of RLNs was ambiguous. A good staging system should meet the following criteria: 1) survival rates should differ among the groups (hazard discrimination); 2) the subsets defined by the T, N, and M classifications that comprise a given group should have similar survival rates (hazard consistency); 3) the patient distribution across the groups should be balanced; and 4) the cure prediction should be high (outcome prediction) [26]. The inclusion of CLN-/RLN + patients into N1 by the 7^th^ AJCC staging system improved the hazard consistency of N1 patients and improved hazard discrimination among N subsets [19,20]. Now the result of our study showed that the involvement of both CLNs and RLNs might also be a prognostic factor for N1 patients. It is reasonable to hypothesis that defining patients with both CLNs and RLNs involvement as a particular subset of N1 (for instance, N1b) might improve the hazard discrimination of the staging system.

However, there were several limitations of this study. Since it was a retrospective study, the distribution of patient number in different groups and subgroups were not well balanced. The treatment modalities used in patients including radiation technique and chemotherapy regimens were not uniform. The total amount of 142 patients analyzed was a relatively small number. Hence, the current finding could only be taken as preliminary. A larger sample of uniform treatment is needed for observing whether differences in the treatment outcomes exist between N1 patients with both RLNs and CLNs involvement and the rest N1 patients, and even between N1 patients with both RLNs and CLNs involvement and N2 patients, thus to conclude whether changes and improvement are needed in the definition of N1 stage of 7^th^ edition AJCC/UICC staging system.

## Conclusions

Our study was the first one focusing on the prognosis of different subsets of NPC patients with N1 disease (7^th^ edition UICC/AJCC staging system). The 5-year OS was 89.8% and the main treatment failure pattern was distant metastasis. The involvement of both cervical lymph nodes and retropharyngeal lymph nodes may be a potentially prognostic factor for distant metastasis and disease progression in N1 patients, which needs a larger sample to confirm the result. The N stage for N1 patients with involvement of both cervical lymph nodes and retropharyngeal lymph nodes may need to be deliberated.

## Competing interests

The authors declare that they have no competing interests.

## Authors’ contributions

QS and CH did study design, data acquisition, data analysis and drafted the manuscript. CS and TX provided patient data and clinical support. LK participated in study design and provided patient data. JD provided MRI images and participated in MRI reading. XW and YG provided patient data and participated in data analysis. All authors read and approved the final manuscript.
